# Prenatal Amnioinfusion as a Diagnostic Tool in Severe Oligo- and Anhydramnios: A Retrospective Single-Center Experience with Descriptive Perinatal Outcomes

**DOI:** 10.3390/jcm15020511

**Published:** 2026-01-08

**Authors:** Kristin Andresen, Christel Eckmann-Scholz, Andre Farrokh, Ulrich Pecks, Nicolai Maass, Veronika Günther, Ibrahim Alkatout, Johannes Ackermann

**Affiliations:** 1 Department of Obstetrics and Gynecology, University Hospital Schleswig-Holstein, Kiel Campus, 24105 Kiel, Germany; christel.eckmann@uksh.de (C.E.-S.); andre.farrokh@uksh.de (A.F.); nicolai.maass@uksh.de (N.M.); ibrahim.alkatout@uksh.de (I.A.); johannes.ackermann@uksh.de (J.A.); 2 Center for Prenatal Diagnostics, University Hospital Schleswig-Holstein, Kiel Campus, 24105 Kiel, Germany; 3 Department of Obstetrics and Gynecology, Institute of Maternal Health and Midwifery, University Hospital Würzburg, 97080 Würzburg, Germany; pecks_u@ukw.de; 4 University Fertility Center, University Hospital Schleswig-Holstein, Kiel Campus, 24105 Kiel, Germany; veronika.guenther@uksh.de

**Keywords:** fetal therapy, ultrasound-based diagnosis, fetal growth restriction, preterm premature rupture of membranes, prenatal imaging, genetic testing in pregnancy, second-trimester complications, perinatal outcome

## Abstract

**Objective**: To evaluate the diagnostic utility of antepartum amnioinfusion in cases of severe oligo- and anhydramnios and to descriptively report perinatal outcomes. **Methods**: This retrospective single-center study analyzed all antepartum amnioinfusions performed between 2009 and 2024 in pregnancies between 16 + 0 and 34 + 0 weeks of gestation. The primary endpoint was diagnostic impact following amnioinfusion. Secondary endpoints were descriptive perinatal outcomes. No inferential statistical analyses were performed. **Results**: A total of 37 amnioinfusions were performed in 31 patients. Median gestational age at first amnioinfusion was 22 ± 4.3 weeks, with a mean infusion volume of 259 ± 59.4 mL. The most frequent etiologies were preterm prelabor rupture of membranes (PROM, 29%), fetal growth restriction (FGR, 25.8%), and urogenital malformations (22.6%). Amnioinfusion improved sonographic visualization and diagnostic assessment in the majority of cases. Pregnancy prolongation was observed in selected subgroups; however, causal inference regarding therapeutic efficacy cannot be drawn. **Conclusions**: Antepartum amnioinfusion represents a valuable adjunct for prenatal diagnostic evaluation in severe oligo- and anhydramnios. Observed perinatal outcomes should be interpreted descriptively. Further prospective, controlled studies are required to define the role of amnioinfusion beyond diagnostic feasibility.

## 1. Introduction

Severe oligo- or anhydramnios in the second trimester is usually caused by preterm prelabor rupture of membranes (PPROM), congenital urogenital tract malformations, placental dysfunction or severe fetal anomalies. These conditions substantially limit ultrasonographic visualization and may impede accurate prenatal diagnosis. Prenatal MRI examination for diagnostic purposes is costly, not always available, and requires sufficient expertise [1,2]. In these cases, amnioinfusion is a useful procedure to enhance chances of diagnosing the underlying cause, given appropriate ultrasound conditions and simultaneous genetic analysis. In rare cases it could also serve as a means of preventing postpartum complications. Some observational studies have shown a lower perinatal mortality rate after amnioinfusion compared to no intervention, whereas the only randomized controlled trial (RCT) to date reported no difference in perinatal mortality and neonatal morbidity [3].

Antepartum amnioinfusion has primarily been proposed as a diagnostic adjunct to improve sonographic conditions and facilitate targeted fetal assessment in pregnancies complicated by severe oligo- or anhydramnios. Previous studies have demonstrated improved visualization of fetal anatomy and enhanced diagnostic confidence following amnioinfusion. Potential therapeutic effects, such as pregnancy prolongation or improvement of perinatal outcomes, have been discussed in the literature; however, evidence remains heterogeneous and inconclusive [4,5,6]. Table 1 shows potential diagnostic and therapeutic improvements achieved through amnioinfusion.

The aim of the present study was to review a long-term single-center experience with antepartum amnioinfusion, focusing on its diagnostic impact and providing a descriptive overview of perinatal outcomes across different etiologies of oligo- and anhydramnios.

## 2. Materials and Methods

### 2.1. Study Design and Population

We conducted a retrospective single-center analysis of all antepartum amnioinfusions performed at our prenatal department between 2009 and 2024. The indication was severe oligo- to anhydramnios determined by the single deepest pocket method between 16 + 0 weeks and 34 + 0 weeks of gestation.

The retrospective data analysis was approved by the local ethics committee (D 506/24). Patients received amnioinfusion after providing their informed consent.

### 2.2. Amnioinfusion Procedure

Under sterile conditions and continuous ultrasound guidance, a 20 G needle was inserted transabdominally and sterile 0.9% sodium chloride solution was infused into the amniotic cavity. Infusion volume was individualized according to gestational age and sonographic requirements.

### 2.3. Diagnostic Assessment

Diagnostic impact of amnioinfusion was assessed retrospectively based on prenatal imaging reports and clinical documentation. Diagnostic change following amnioinfusion was categorized as: 1. Diagnosis unchanged, 2. Diagnosis refined, or 3. Diagnosis newly established after amnioinfusion.

### 2.4. Outcome and Statistical Analysis

The primary endpoint was diagnostic impact. Secondary endpoints were descriptive perinatal outcomes, including pregnancy duration after amnioinfusion and neonatal course. Given the small sample size, heterogeneity of etiologies, and retrospective design, statistical analysis was limited to descriptive measures (means, medians, standard deviations, and frequencies). No inferential statistical analyses were performed.

## 3. Results

### 3.1. Patient Characteristics

**Figure 1:**
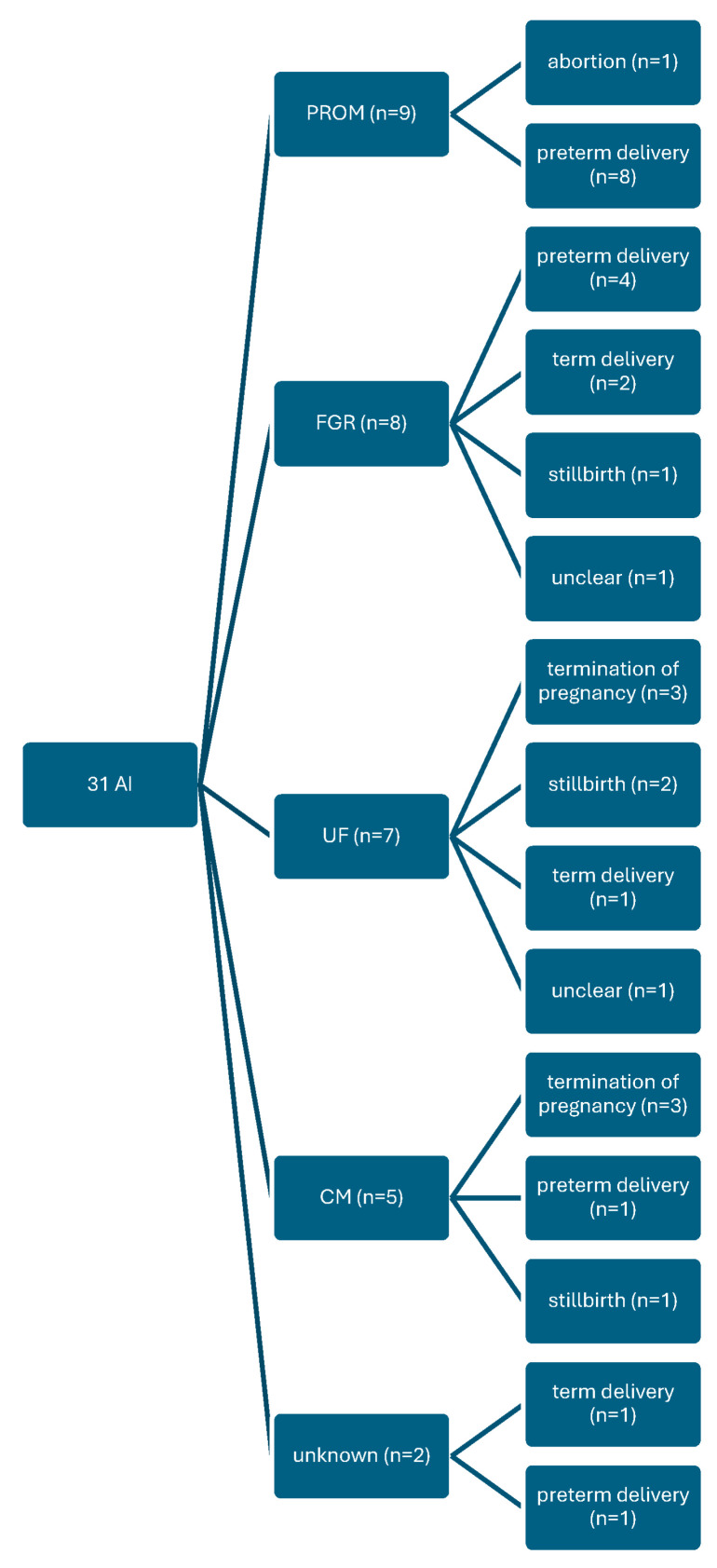
Amnioinfusion, subgroup analysis; AI: amnioinfusion; PROM: preterm rupture of the membranes; FGR: fetal growth restriction; UF: urogenital malformation; CM: chromosomal aberration/severe malformation.

A total of 37 amniotic fluid infusions were administered in 31 patients. The amnioinfusion was carried out at a median time point of 22 ± 4.3 weeks of gestation, with 259 ± 59.4 mL of fluid infused. Etiologies of oligo- or anhydramnios included premature ruptur oft he mebranes (PROM) (n = 9), fetale growth restrivtion (FGR) (n = 8), urogenital malformations (n = 7), chromosomal aberrations or severe malformations (n = 5), and unknown causes (n = 2). Figure 1 shows the distribution of cases according to the various causes of oligohydramnios with the clinical outcomes.

### 3.2. Diagnostic Impact

Amnioinfusion improved sonographic visualization in the majority of cases, allowing more detailed assessment of fetal anatomy. Diagnostic refinement or new diagnostic information was achieved across all etiologic subgroups, particularly in cases of suspected structural anomalies and FGR, where exclusion of additional malformations and clarification of underlying pathology were possible.

### 3.3. Outcomes by Etiology

#### 3.3.1. PROM

In PROM cases, median pregnancy prolongation after first amnioinfusion was 8 ± 3.3 weeks. Perinatal outcomes were heterogeneous, ranging from favorable neonatal courses to adverse outcomes, including neonatal death. Of the children born after PROM, four fetuses showed a good perinatal outcome over time. In this group, three children died, one child had an unfavorable course with hydrocephalus/microcephaly, and one child had a respiratory distress syndrome grade 2 with a tonus regulation disorder. No causal relationship between amnioinfusion and pregnancy prolongation can be inferred.

#### 3.3.2. Fetal Growth Restriction

**Figure 2:**
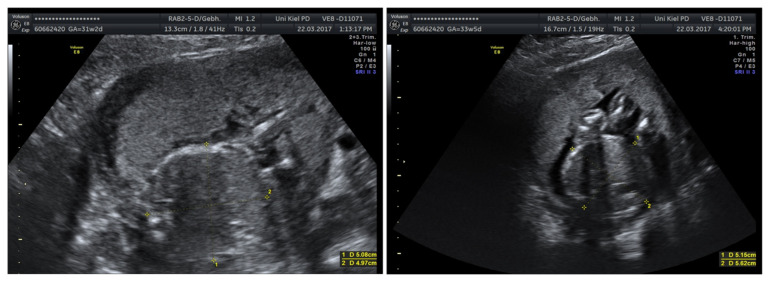
Amnioinfusion in a case of severe fetal growth restriction with anhydramnios before (**left**) amnioinfusion (less movement), and after (**right**) amnioinfusion (increased intrauterine fetal movement).

In cases of fetal growth restriction, pregnancy was prolonged by a mean of 6 ± 6.2 weeks, indicating substantial interindividual variability. Improved Doppler findings were observed in some cases following amnioinfusion. Outcomes varied, including both favorable neonatal survival and intrauterine fetal death. Figure 2 shows amnioinfusion in a case of severe fetal growth restriction with anhydramnios before (left) and after (right) amnioinfusion.

#### 3.3.3. Urogenital Malformations

**Figure 3:**
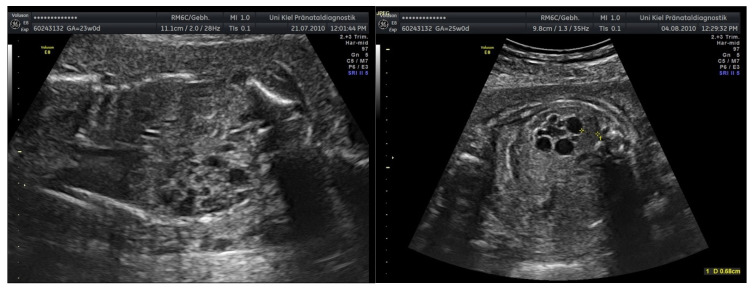
Multicystic renal degeneration before (**left**) and after (**right**) amnioinfusion.

In cases of urogenital malformations, amnioinfusion primarily facilitated improved diagnostic visualization and genetic clarification. Among children with urogenital malformations, pregnancy was terminated upon request in three cases. One child had multicystic renal degeneration (Figure 3, one had additional malformations, and one child had uropathy. Two children experienced IUFT (intrauterine fetal death). One child had an uneventful course. One mother started to attend a different clinic and was not accessible to further investigation. Figure 3 shows amnioinfusion in a case of multicystic renal degeneration before (left) and after (right) amnioinfusion. Neonatal outcome was determined by the underlying malformation.

#### 3.3.4. Chromosomal Aberrations and Severe Malformations

**Figure 4:**
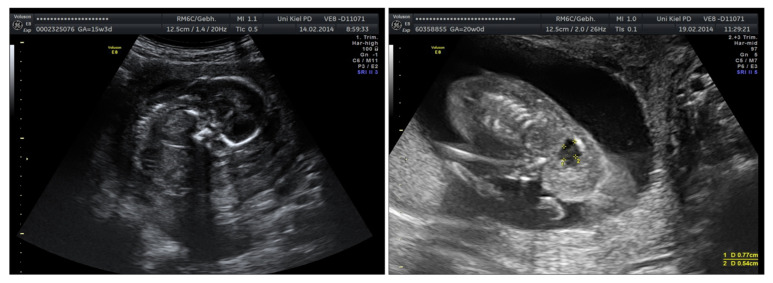
Caudal regression syndrome before (**left**) and after (**right**) amnioinfusion.

**Figure 5:**
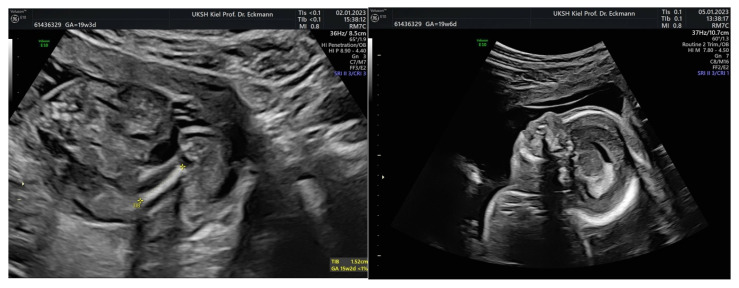
Fetus with a severe malformation before amnioinfusion (**left**); the same fetus shows a clearly visible severe hyperflexion of the cervical spine (**right**).

Among fetuses with chromosomal aberrations or severe malformations (Figure 4 and Figure 5), one fetus had IUFT, pregnancy was terminated upon request in three cases, and one fetus with VACTERL association underwent secondary cesarean section at 23 + 5 weeks due to amniotic infection syndrome. Figure 4 and Figure 5 show the improved visibility after amnioinfusion in cases of complex malformations.

In fetuses with chromosomal abnormalities or severe malformations, amnioinfusion improved visualization but did not alter prognosis. Outcomes included pregnancy termination, intrauterine fetal death, and extreme preterm delivery.

## 4. Discussion

Amnioinfusion is a diagnostic and potentially therapeutic tool in prenatal diagnostics for patients with oligo- to anhydramnios. However, we are still not certain about the specific cases that benefit in terms of improved diagnosis or even an improvement of pregnancy outcomes. In addition, the potential risks for both mother and child have been rated differently in the recently published literature.

The aims of this study were: (a) to review the amnioinfusions performed at our unit, (b) draw conclusions about the causes of pronounced oligo- to anhydramnios and (c) investigate the risks and potential benefits of the procedure. We focused on the postnatal course and postnatal outcomes, as these have not been addressed in detail in the published literature.

### 4.1. Diagnostic Value

We observed a substantial improvement in diagnostic accuracy across all cases. The enhancement applied not only to the exclusion of fetal anomalies, but also to the more precise characterization of detected fetal anomalies. The effectiveness of improving diagnostic precision through this approach has been demonstrated in numerous prior studies. Gembruch et al. [5] showed that the infusion of amniotic fluid not only enhances ultrasonographic conditions, but also permits a clearer differentiation between fetal renal dysfunction and fetuses with intrauterine growth restriction (FGR). These data are further corroborated by the findings of Fisk et al. [4] and Quetel et al. [6], who also reported significant improvements in the clarity and accuracy of fetal anomaly detection using similar methods. In view of the fact that fetuses with oligohydramnios are more frequently affected by malformations [7], we found that the diagnosis of fetal renal agenesis and sirenomelia was improved by the use of Doppler ultrasound examinations [8]. These examinations provide crucial information, but their diagnostic reliability is highly dependent on the position of the fetus within the uterus, as highlighted by Heyl et al. [9,10]. This dependency underscores the importance of optimizing fetal position in order to maximize the utility of Doppler imaging. In particular, amnioinfusion improved diagnostic capabilities by providing better ultrasound conditions due to its contrast-enhancing properties as a physiological medium [10]. In certain clinical situations, a supplementary magnetic resonance imaging (MRI) examination might be beneficial as it offers additional insights that ultrasound alone might not reveal. However, it should be noted that MRI is significantly expensive by comparison and requires more extensive resources and time, as reported by Levine et al. [1]. Taking into account the outcomes reported in these previous investigations, along with our findings, it becomes evident that amnioinfusion constitutes a significant advancement in the field of prenatal diagnostics. The technique not only enhances the accuracy of identifying and classifying fetal anomalies, but also supports informed decision-making in the clinical management of pregnancies with suspected fetal anomalies. The comprehensive effect of amnioinfusion in terms of improving the diagnostic landscape allows for better preparation and management of potential complications during pregnancy, as reported by Vikraman [10].

Ultimately, the collective evidence suggests that amnioinfusion is a valuable tool in the arsenal of prenatal diagnostic techniques. It contributes significantly to the refinement of diagnostic processes, providing both clinicians and expectant parents with clear and reliable information for informed decision-making. This reinforces the potential role of amnioinfusion in supporting high-quality prenatal care.

### 4.2. Safety Aspects and General Outcomes

In our study, the median duration of pregnancy after an amnioinfusion was 8 ± 3.3 weeks. None of the patients experienced complications attributable to amniotic fluid infusion leading to pregnancy loss, which is consistent with the data reported by Vikraman et al. [10,11]. These findings show that amnioinfusion, performed by an experienced practitioner, is a safe tool in prenatal diagnostics and can be offered to pregnant women when indicated.

### 4.3. Preterm Rupture of the Membranes

Our data revealed that PROM is one of the most common causes of oligo- or anhydramnios, and that a prolongation of pregnancy was achieved in patients with PROM. However, the extent to which the prolongation was facilitated by amnioinfusion cannot be conclusively stated due to the absence of a control group. Moreover, reductions in perinatal mortality and morbidity are not apparent from the data.

A report published in 2014 by Hofmeyr [12] showed that amnioinfusion after PROM is associated with reduced rates of neonatal death, neonatal sepsis, and pulmonary hypoplasia. In addition, amnioinfusion was found to reduce the likelihood of delivery within the first 7 days after PROM. This suggests that amnioinfusion may play a role in prolonging pregnancy and improving neonatal outcomes in cases of PROM [13]. However, the evidence remains mixed.

Roberts et al. [14] compared serial weekly transabdominal amnioinfusions with expectant management until 37 weeks’ gestation in cases of PROM occurring between 16 and 24 + 0 weeks of pregnancy. Their findings showed no significant difference in outcomes between the two groups, but the sample size in their study was too small to draw definitive conclusions. This highlights the need for further research in larger sample sizes in order to better understand the potential benefits of amnion infusion.

In our investigation, as well as in other studies, labor did not immediately follow membrane rupture when accompanied by amnioinfusion. This fact could suggest that prolongation of pregnancy is possible. One hypothesis is that the infusion of amniotic fluid may help flush out inflammatory products and bacteria, which might play a role in delaying labor [15].

Previous data, such as those from van Kempen et al. [16], have not demonstrated a significant reduction in perinatal morbidity and mortality with amnioinfusion. Although the initial findings of Tchirikov et al. have been published [17], the complete analysis is yet to be released. Therefore, conclusions regarding long-term efficacy and safety remain preliminary. The above mentioned investigations may provide additional insights into the potential long-term benefits of amnioinfusion, particularly in managing complex cases of PROM and fetal growth restriction. The studies may help to clarify whether amnioinfusion can consistently improve pregnancy outcomes and provide clear guidelines for clinical practice.

### 4.4. Fetal Growth Restriction

The second most common cause of oligohydramnios is fetal growth restriction (FGR). We identified eight cases of FGR, each showing pathological findings in the uterine and/or umbilical Doppler ultrasound. By the use of amnioinfusion we were able to exclude further pathologies. Additionally, a simultaneous amniotic fluid analysis ruled out chromosomal and syndromic conditions, which are more frequently observed in FGR fetuses [18].

**Figure 6:**
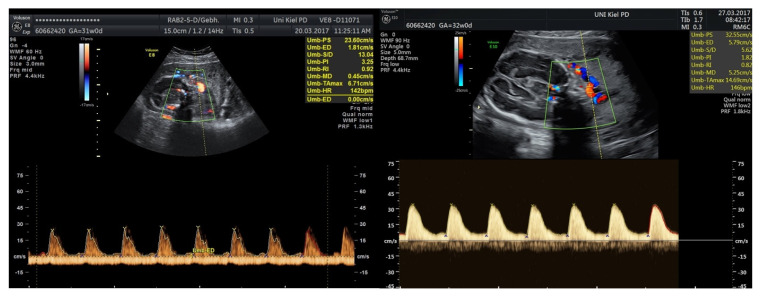
Umbilical Doppler showing absent end-diastolic flow before amnioinfusion (**left**), and positive end-diastolic flow (**right**) four days after amnioinfusion.

Takahashi [19] performed amnioinfusion in cases of FGR fetuses presenting with pathological Doppler findings or CTG decelerations. The study demonstrated an improvement in Doppler pathology and a significant prolongation of pregnancy, extending the gestational period by at least 4 weeks in 78% of cases. Similarly, we observed a prolongation of pregnancy after the use of amnioinfusion, although the duration of the prolongation varied considerably, ranging from a minimum of 12 days to as long as 15 weeks in the FGR subgroup. We also registered an improvement in umbilical Doppler findings after amnioinfusion (Figure 6). However, unlike Takahashi et al. [19], we did not administer tocolysis in any case.

Further evidence supporting the potential benefits of amnion infusion in the FGR subgroup can be found in individual case reports by Sarno and Katsura; [20,21] these reports suggest that amnioinfusion may help prolong pregnancy and improve umbilical cord blood flow. Studies with limited case numbers showed that amnioinfusion is able to effectively alleviate repetitive variable decelerations in the first stage of labor [22,23]. Moreover, Butt et al. [24] reported that, even in cases of isolated oligohydramnios, pregnancies could be prolonged by amnioinfusion. The prolongation of pregnancy may reduce morbidity and mortality, particularly in cases of preterm births. However, as in our study, a control group was absent in the afore-mentioned investigation, underscoring the need for additional research in this area to further substantiate these findings.

### 4.5. Urogenital Malformations and Chromosomal Abnormalities

The third group with severe oligo- to anhydramnios consisted of urogenital malformations and other major syndromes. In these cases, amnioinfusion primarily enhanced the information provided by diagnostic ultrasound and clarified genetic data. An improvement in neonatal outcome is not expected here, as the underlying disease prior to the oligo- and anhydramnios determines the prognosis. This is further highlighted by the significantly higher rate of pregnancy termination in this group.

### 4.6. Technical Aspects of Amnioinfusion Fluids

We used normal saline (NaCl 0.9%) because of its wide availability, safety profile, and extensive clinical experience supporting its use in amnioinfusion. It is isotonic and poses a minimal risk of electrolyte imbalance, making it a practical and effective option in most clinical settings. While it does not perfectly mimic the composition of natural amniotic fluid, studies have demonstrated its efficacy in reducing variable decelerations and improving fetal outcomes during labor. Table 2 outlines additional options for amnioinfusion, highlighting their composition, clinical benefits, potential risks, and current evidence.

### 4.7. Therapeutic Considerations and Emerging Approaches

In recent years, antepartum amnioinfusion has also been discussed in the context of emerging fetal therapeutic strategies. In contrast to its established diagnostic use, these approaches aim to restore or maintain amniotic fluid volume over a prolonged period through serial amnioinfusion, particularly in cases of early-onset anhydramnios caused by severe renal anomalies. The underlying biological rationale is that sustained restoration of amniotic fluid may support pulmonary development during critical phases of fetal growth and thereby mitigate pulmonary hypoplasia.

Proof-of-concept data for such therapeutic strategies have been reported by highly specialized fetal therapy centers. In particular, the Renal Anhydramnios Fetal Therapy (RAFT) trial demonstrated that serial amnioinfusion may enable postnatal survival in selected fetuses with otherwise lethal bilateral renal agenesis, albeit with substantial neonatal morbidity and the need for intensive postnatal care [26].

Related serial amnioinfusion strategies have also been investigated by groups at specialized centers such as Johns Hopkins University, focusing on procedural feasibility, infusion protocols, and maintenance of amniotic fluid volume in highly selected patient populations [27]. These studies were conducted within predefined interventional frameworks, involved repeated procedures, and targeted specific therapeutic endpoints, most notably pulmonary development.

Importantly, these experimental therapeutic approaches differ fundamentally from the design and intent of the present study. Our cohort was heterogeneous with respect to etiology, serial amnioinfusion protocols were not applied, and therapeutic pulmonary outcomes were not predefined endpoints. Consequently, findings from serial amnioinfusion studies conducted within fetal therapy programs cannot be extrapolated to the perinatal outcomes observed in our population. The aforementioned studies are therefore discussed here solely to contextualize ongoing research developments in the field of fetal therapy and should not be interpreted as evidence of therapeutic efficacy of antepartum amnioinfusion within the scope of this retrospective diagnostic study.

### 4.8. Limitations

Several limitations of this study must be acknowledged. First, the retrospective single-center design limits generalizability and precludes causal inference regarding diagnostic or therapeutic effects of antepartum amnioinfusion. Second, the overall sample size was small, and subgroup analyses were further limited by pronounced heterogeneity of underlying etiologies, including PROM, fetal growth restriction, urogenital malformations, and chromosomal or syndromic abnormalities. These conditions differ substantially in pathophysiology, prognosis, and clinical course, which restricts direct comparability of outcomes across groups.

Third, no control group without amnioinfusion was available, and inferential statistical analyses were therefore not performed. All outcome measures should be interpreted descriptively. Observed pregnancy prolongation in selected cases may reflect individual disease trajectories and concurrent clinical management rather than a direct effect of amnioinfusion.

In addition, the long inclusion period spanning 2009 to 2024 represents an important limitation. During this time, significant advances occurred in ultrasound technology, prenatal genetic diagnostics, neonatal intensive care, and the clinical management of conditions such as PROM and fetal growth restriction. These temporal changes may have influenced both diagnostic assessment and perinatal outcomes and introduce a potential time-span bias.

Finally, diagnostic improvement was assessed retrospectively based on documented ultrasound findings and clinical reports rather than through predefined prospective criteria. Although this reflects real-world clinical practice, it may have introduced observer-dependent variability and limits standardization of diagnostic endpoints.

## 5. Conclusions

Antepartum amnioinfusion was feasible and safely performed in this retrospective cohort of pregnancies complicated by severe oligo- or anhydramnios. In all cases, restoration of amniotic fluid volume allowed improved ultrasound visualization, which facilitated a more detailed assessment of fetal anatomy and supported diagnostic clarification.

Perinatal outcomes varied widely and were primarily determined by the underlying etiology, particularly in cases involving severe malformations or chromosomal abnormalities. While pregnancy prolongation was observed in selected cases, especially in PROM and fetal growth restriction, the absence of a control group and the heterogeneity of the cohort preclude conclusions regarding therapeutic efficacy.

Overall, the results support the use of antepartum amnioinfusion as a diagnostic adjunct in complex pregnancies with markedly reduced amniotic fluid volume. Further prospective studies with standardized diagnostic endpoints and etiology-specific analyses are required to define its role beyond diagnostic assessment.

## Figures and Tables

**Table 1:** Potential diagnostic and therapeutic improvements achieved through amnioinfusion.

Diagnosis	Treatment
**Further human genetic examination**	Prevention of pulmonary hypoplasia and skeletal deformation
**Improvement of ultrasonographic conditions**	Decompression of the umbilical cord
	Improving postnatal outcomes

**Table 2:** Fluids used for amnioinfusion [17,22,25].

Fluid	Composition	Advantages	Disadvantages	Clinical Application	Evidence/Studies
NaCl 0.9% (Normal Saline)	Isotonic NaCl (154 mmol/L Na^+^, 154 mmol/L Cl)	Widely available, inexpensive, safe to use.	No buffering, lacks other electrolytes, not physiologically equivalent to amniotic fluid.	Standard for intrapartum amnioinfusion, especially in cases of variable decelerations.	Several RCTs support efficacy in reducing variable decelerations. (PMID: 2187344) [22].
Ringer’s lactate	Na^+^, K^+^, Ca^2+^, Cl, lactate (closer to amniotic fluid)	More physiological electrolyte profile, buffered.	Slightly more expensive, contraindicated in hypercalcemia.	Alternative to saline, often used in prolonged infusion or oligohydramnios.	Comparable efficacy to saline (ScienceDirect: S0002937896800299) [25].
Sterile water	Hypotonic, no electrolytes.	Occasionally usable in emergencies.	Risk of electrolyte shifts, edema; hypotonic—potentially harmful.	Not recommended; emergency or diagnostic use only.	Described in experimental contexts, not for routine use.
Ringer + Glucose	Ringer‘s solution with 5% glucose	Combines electrolytes and energy.	Risk of maternal hyperglycemia or fetal osmolar shifts.	Experimental; can be used as combined maternal-fetal therapy.	Anecdotal reports only, not established.
Amnion Flush Solution	Commercially prepared sterile solution designed to mimic natural amniotic fluid; varies by manufacturer.	Closer biochemical similarity to natural amniotic fluid; sterile and standardized.	Availability may be limited; more expensive than standard fluids.	Used in selected clinical centers for flushing or infusion, especially in therapeutic amnioinfusion.	Limited data; some reports support improved fetal visualization and comfort during intrauterine procedures [19].

## Data Availability

The data presented in this study are available on request from the corresponding author. The data are not publicly available due to ethical restrictions and patient confidentiality.
